# Assessing Omega-3 Therapy and Its Cardiovascular Benefits: What About Icosapent Ethyl? A Systematic Review and Meta-Analysis

**DOI:** 10.3390/ph18040601

**Published:** 2025-04-20

**Authors:** Nathália Mendes Machado, Maria Vitória Barroso Oliveira, Karina Quesada, Jesselina Francisco dos Santos Haber, Ricardo José Tofano, Claudio José Rubira, Tereza Lais Menegucci Zutin, Rosa Direito, Eliana de Souza Bastos Mazuqueli Pereira, Camila Marcondes de Oliveira, Ricardo de Alvares Goulart, Vitor Engrácia Valenti, Kátia Portero Sloan, Lance Alan Sloan, Lucas Fornari Laurindo, Sandra Maria Barbalho

**Affiliations:** 1 Department of Biochemistry and Pharmacology, School of Medicine, University of Marília (UNIMAR), Marília 17525-902, São Paulo, Brazilricardogoulartmed@hotmail.com (R.d.A.G.); lucasffffor@gmail.com (L.F.L.); 2 Postgraduate Program in Structural and Functional Interactions in Rehabilitation, University of Marília (UNIMAR), Marília 17525-902, São Paulo, Brazil; 3 Laboratory of Systems Integration Pharmacology, Clinical and Regulatory Science, Research Institute for Medicines, Universidade de Lisboa (iMed.ULisboa), Av. Prof. Gama Pinto, 1649-003 Lisbon, Portugal; 4 Faculty of Philosophy and Sciences, Universidade Estadual Paulista (UNESP), Marília 17525-900, São Paulo, Brazil; 5 Department of Clinical Metabolism, Texas Institute for Kidney and Endocrine Disorders (TIKED), Lufkin, TX 75904, USA; 6 Department of Internal Medicine, University of Texas Medical Branch, Galveston, TX 77555, USA; 7 Research Coordination, UNIMAR Charitable Hospital, Marília 17525-902, São Paulo, Brazil

**Keywords:** Icosapent ethyl, eicosapentaenoic acid, EPA, lipid-lowering therapies, lipids, cardiovascular diseases

## Abstract

**Background:** Lipid-lowering therapies are an option for stabilizing lipid levels. Icosapent ethyl (IPE) is a highly purified formulation of eicosapentaenoic acid, which can reduce lipid action, improve plaque stabilization, reduce platelet aggregation, lower TG, and prevent cardiovascular events. IPE is frequently used with statins to manage elevated TG levels. However, the evidence on IPE as a lipid-lowering agent is limited, and no updated systematic review and meta-analysis have been published considering the recent advancements in the field and newly published studies. Therefore, we aim to fill this gap. **Methods:** We used the PRISMA guidelines and the PICO (Population, Intervention, Comparison, and Outcome) framework to conduct this review, aiming to answer the question, “Can IPE benefit patients at cardiovascular risk?” GRADE was used to evaluate evidence levels to adhere to the highest criteria. **Results:** Predominantly, the evaluated population presented TG levels between ≥135 mg/dL and 500 mg/dL and LDL-C levels between >40 mg/dL and ≤100 mg/dL. The included studies showed a reduction in TG and LDL-C and a decrease in cardiovascular events. It means that, according to our systematic review evidence analysis, IPE has been effective in lowering blood lipid levels, including TG, and reducing cardiovascular death and events, such as non-fatal stroke or hospitalization for unstable angina. However, it is worth noting that these results were primarily from patients undergoing statin therapy. According to our meta-analysis, IPE may not be considered a lipid-lowering drug, as limited action associated with its use was evident in the quantitative results. However, caution is necessary, as only two studies were suitable for inclusion due to the differing outcomes in the analyzed samples. **Conclusions:** Despite the quantitative synthesis, IPE possesses anti-inflammatory, anti-thrombotic, and anti-atherogenic properties, highly related to cardiovascular protection. Based on our included studies, IPE was considered a promising therapy for atherosclerotic cardiovascular disease in conjunction with other lipid-lowering therapies, particularly statins, for patients with extremely high TG levels. The limitations of the reviewed studies may include small sample sizes, varying outcomes, and a small duration of interventions. Future clinical trials with similar outcomes, sample sizes, and intervention durations must be designed, and updated meta-analyses must be published in the following years to fully assess the effects of IPE as a lipid-lowering and cardiovascular protector drug.

## 1. Introduction

Cardiovascular diseases (CVD) are the leading cause of death worldwide. Among these diseases, cerebrovascular accidents and atherosclerotic cardiovascular disease (ASCVD), which have age, family history, diabetes mellitus (DM), high levels of low-density lipoprotein-cholesterol (LDL-C), and high triglyceride (TG) levels as risk factors, are the most prevalent ones [1,2,3,4,5,6,7,8].

CVD are closely associated with high serum concentrations of lipoproteins and TG; therefore, reducing LDL-C and TG levels reduces cardiovascular risk [6,9,10,11]. Lipid-lowering therapies (LLT) were established to control lipid levels, and the available options currently include statins, ezetimibe, omega-3 (ω-3) supplements, and protein convertase subtilisin/kexin type 9 (PCSK9) inhibitors, used to prevent and improve the prognosis of CVD [4,5,12,13,14].

The main LLT are statins, considered essential drugs for reducing lipid levels; however, combined therapy with statins and other lipid-lowering drugs (LLD) has brought several benefits, especially for patients at a high risk of ASCVD [15,16,17].

**Figure 1:**
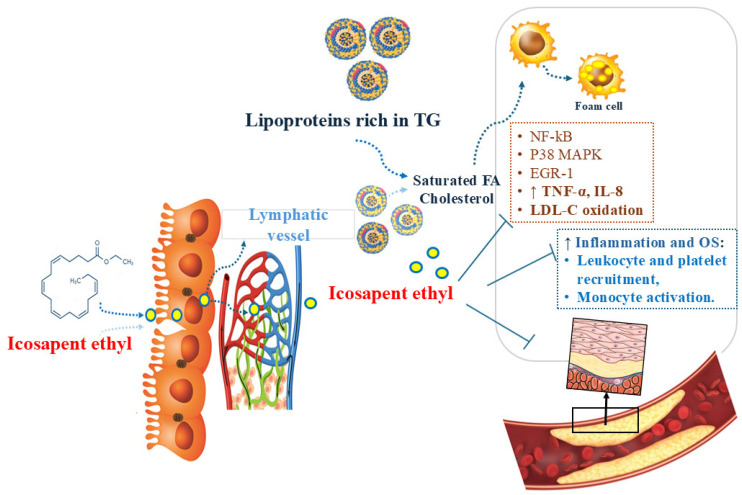
Structure, absorption, and distribution of Icosapent Ethyl. EGR-1: Early growth response 1; FA: Fatty acid; IL: Interleukin; LDL-C: Low-density lipoprotein cholesterol; MAPK: Mitogen-activated protein kinase; NF-kB: Nuclear factor KB; OS: Oxidative stress; TG: Triglycerides; TNF-α: Tumor necrosis factor-α.

Icosapent ethyl (IPE) is a formulation of eicosapentaenoic acid (EPA) with a 99.99% purity, which means a very low concentration of ω-3 fatty acid docosahexaenoic acid (DHA). EPA consists of a ω-3 polyunsaturated fatty acid (PUFA) obtained from fish oil, presenting a chemical structure of a 20-carbon fatty acid containing five double bonds, with the first one located at the third carbon atom from the distal end of the compound [1,18,19,20,21,22]. Currently, the two highly purified formulations of IPE available on the market are Vascepa^®^ and Epadel^®^ [1,23,24]. Figure 1 shows the structure, absorption, and distribution of IPE.

EPA promotes several beneficial effects in the cardiovascular system, such as reduced lipid action, plaque stabilization, antiproliferation, vasodilation, and reduction in platelet aggregation, lower TG and non-high-density lipoprotein cholesterol (non-HDL-C) levels, and improved endothelial function. It prevents cardiovascular events such as myocardial infarction (MI), coronary revascularization, strokes, and unstable angina [1,25]. In addition, EPA has antioxidant and anti-inflammatory effects that reduce the accumulation of macrophages, thereby reducing the accumulation of foam cells and improving monocyte adhesion and endothelial function [26,27].

IPE is primarily associated with statin therapy for patients with established CVD, hypertriglyceridemia, and DM to enhance treatment and prevent cardiovascular damage. EPA is the first medication in its class to be approved for use. In a systematic review published in 2022, the authors concluded that existing trials have demonstrated that Vascepa^®^ reduces TG levels and the risk of CVD in patients receiving statin therapy [28,29].

Despite extensive discussion following large-scale studies on the effects of IPE against dyslipidemia in various populations, its use is not free of being associated with some discrepancies. IPE’s effects as lipid-lowering remain obscure in some aspects of specific population settings, including those heart diseases that require strict control of heart physiology. Notably, the REDUCE-IT and STRENGTH trials are the most prominent clinical studies that assessed the effects of IPE as an antilipidemic; contradictory results are derived from these trials [30,31]. Both results sparked debates regarding whether IPE might be an effective strategy to mitigate cardiovascular risk. The REDUCE-IT trial demonstrated that IPE significantly reduces major adverse cardiovascular events, with a primary outcome occurring in 17.2% of the treated group versus 22.0% in the placebo group. However, the STRENGTH trial found that the remedy does not reduce cardiovascular outcomes, with the primary outcome occurring in 12.0% of the treated group versus 12.2% in the placebo group. Differences in active oils in the utilized supplements and comparator oils might be an explanation [32]. Overall, in the IPE studies, up to 45.0% of patients were eligible to receive the medications, including the REDUCE-IT trial, the US Food and Drug Administration, and the Health Canada label. Despite the abovementioned discrepancies, IPE has been considered cost-effective compared to standard care across various countries, including the United States, Canada, Germany, Israel, and Australia [33].

Due to the exponential growth in CVD, the relevance of studies on drugs that mitigate risk factors is essential. Therefore, this systematic review aimed to demonstrate the effects of IPE on the therapeutic approach to plasma lipids in cardiovascular patients and perform a meta-analysis of the eligible studies. This review differs from existing ones, encompassing all the published clinical trials. In addition, it shows the effects of IPE compared to other LLD.

## 2. Materials and Methods

### 2.1. Focused Question

This systematic review was built to unravel the question: Can Icosapent Ethyl benefit patients at cardiovascular risk?

### 2.2. Language

Only trials published in English were included.

### 2.3. Literature Search and Databases

The literature search investigated studies in the COCHRANE, EMBASE, and MEDLINE–PubMed databases. The mesh terms used were “Icosapent ethyl” and “Total cholesterol” or “TG” or “LDL-C” or “VLDL-C” or “HDL-C” or “Metabolic syndrome” or “Cardiovascular risk” or “Cardiovascular disease” or “Lipids”.

All articles acknowledged were exported to the Rayyan QCRI program (Qatar Computing Research Institute, Qatar) to eliminate duplicates. The Rayyan program screened the studies by reading the title and abstract. By reading all the articles, S.M.B., N.M.M., and K.Q. completed the suitability stage. Another reviewer (R.J.T.) was invited to provide a decision in the event of a disagreement concerning a study. After incorporating the final references, we evaluated the possibility of conducting a meta-analysis within the group. There is no registration number for this systematic review and meta-analysis.

### 2.4. Study Selection

The studies originated from peer-reviewed journals, published from the beginning of the database until August 2024. The inclusion and exclusion criteria agreed with the PICOS (Population, Intervention, Comparison, Outcomes, and Study Design) elements, including the following:

(P) Patients older than 18 years old;
(I) The intervention group ought to receive IPE;
(C) For comparison groups, we included studies that evaluated subjects who received a placebo;
(O) The outcome of interest was total cholesterol or TG or LDL-C or very low-density lipoprotein (VLDL) or high-density lipoprotein cholesterol (HDL-C);
(S) We included studies with no blinding, single-blinded, or double-blinded randomized control and crossover designs. This investigation was limited to articles published in peer-reviewed journals and written in English. We excluded conference papers, master’s dissertations, doctoral theses, descriptive studies, case studies, editorials, and reviews. The time range for the studies included the years between 1999 and 2024.

### 2.5. Data Extraction

Data concerning the author, study design, features of the study participants, intervention, and exercise protocols of the respective studies were extracted from the primary studies and presented in a table. Missing data were requested by contacting the corresponding study authors. This stage was finished independently by at least two reviewers. When the author’s correspondent did not respond, Web Plot Digitizer^®^ was applied to extract the data presented in graphs. The cardiovascular data were charted as the mean and standard deviation (SD). Values presenting with “standard error” or “confidence intervals” (CI) in the primary studies were converted to SD.

### 2.6. Search and Selection of Relevant Articles

**Figure 2:**
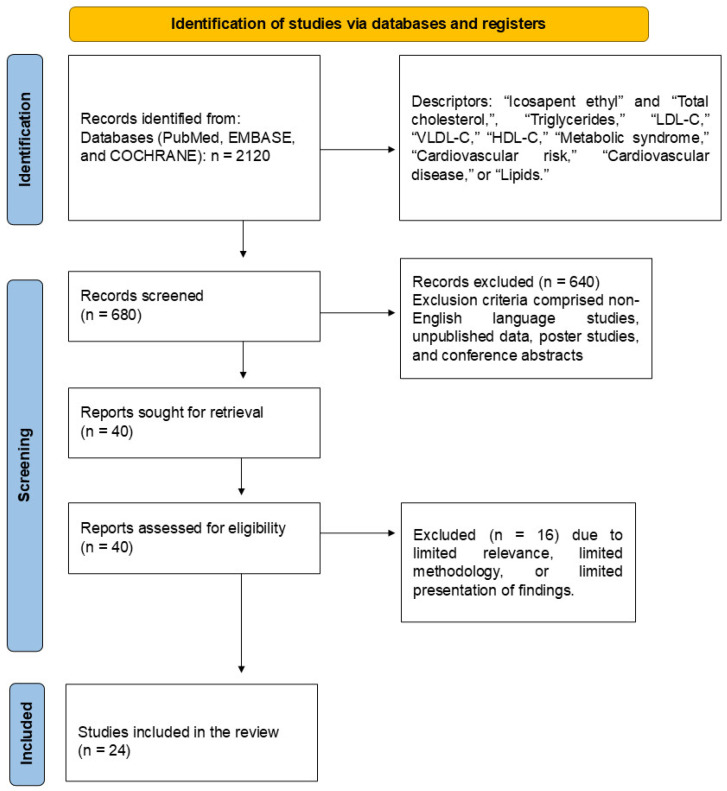
A flow diagram illustrates the study selection process, adhering to PRISMA guidelines.

The study selection was conducted according to the Preferred Reporting Items for Systematic Reviews and Meta-Analyses (PRISMA) guidelines (Figure 2) [34,35].

### 2.7. Data Items

We collected TG, LDL-C, HDL-C, and total lipids data to compare outcomes before and after the intervention. Data related to participant and intervention profiles and funding sources were obtained from the selected references. Missing or unclear information was discarded.

### 2.8. Quality Assessment

The *Cochrane Handbook* was consulted to evaluate potential bias risks associated with the selected trials [36].

### 2.9. Assessment of the Risk of Bias in Individual Studies and Across Studies

The bias analysis was conducted using the risk of bias tools developed by the Cochrane organization [37] through the Review Manager program (RevMan 5.4.1). Risk of bias is a tool based on domains. Its evaluation is split into six areas: “Randomization process”, “Deviations from intended interventions”, “Missing outcome data”, “Measuring of the outcome”, “Selection of the reported results”, and “Overall bias”. The cataloging was split into three categories: low risk, some concerns, and high risk. Two independent authors completed the risk of bias analysis. A further researcher was referred to if there were any inconsistencies in their verdicts. We identified variables that could influence cumulative evidence, such as publication bias and selective reporting within studies. The assessors of the risk of bias were trained in appropriate sessions.

### 2.10. Certainty Assessment (Levels of Evidence)

We used the Grades of Recommendation, Assessment, Development, and Evaluation (GRADE) Working Group (GRADE Working Group, 2004) to gauge the certainty of the evidence. This analysis comprises the study design of the randomized trials (strong evidence). We also considered the study quality, including detailed study methods and execution, and limitations in the strength of evidence analysis [38]. We employed GRADEpro GDT v4^®^ (McMaster University, Hamilton, ON, Canada) to elaborate on the summary of the findings table.

### 2.11. Qualitative Analysis (Systematic Review)

A narrative synthesis was conducted to provide a detailed description of how each study was completed. The details of each study were introduced in texts and tables. The results of the individual qualitative analyses for each study were obtained by analyzing cardiovascular parameters for both the intervention and control protocols.

### 2.12. Synthesis of Results and Summary Measures

After selecting all the references, we evaluated the possibility of a meta-analysis. In favorable situations, we introduced lipid profile values. The information required to construct the meta-analysis included the post-intervention period in both groups. Only references that provided the mean or median and the SD or standard error were included. We adopted the criterion of extracting all data provided between protocols in the post-intervention period.

Heterogeneity was calculated via the I^2^ statistic. We interpreted it as 0–40%: unimportant; 30–60%: moderate heterogeneity; 50–75%: substantial heterogeneity; 75–100%: considerable heterogeneity [39,40]. For the “95% CI” and “test for overall effect size” values, significant differences were assumed for *p*-values less than 0.05 (or 5%). If the studies did not provide dispersion values of change, such as SD, 95% CI, standard errors, or *p*-values, the missing SD of the changes (SD changes) was calculated. The meta-analysis outcomes were reported as a weighted mean difference (MD), 95% CI, and *p*-value. A *p*-value of less than 0.05 was considered statistically significant for the overall MD between the intervention and control groups. The results were presented in forest plots. We employed a random-effects model, a more conservative method that allows study heterogeneity to deviate beyond chance, thereby providing more generalizable results [41]. All data were formed using the Review Manager Program (RevMan 5.4.1).

## 3. Results

The selection of the twenty-four clinical trials included in this review is shown in Figure 2, and the results of these studies are shown in Table 1. The bias risk is shown in Table S1 within the Supplementary Materials. In summary, the included trials evaluated the following conditions: TG level reductions (*n* = 5), total cholesterol reduction (*n* = 1), inflammatory biomarkers such as oxidized-LDL-C (ox-LDL-C) and reactive oxygen species (ROS) (*n* = 3), cardiovascular events (*n* = 7), population with coronary revascularization (*n* = 2), plaque attenuation (*n* = 3), plaque mensuration by coronary computed tomographic angiography (CCTA)/multidetector computed tomography (MDCT)/cardiovascular magnetic resonance (CMR) (*n* = 3), percutaneous coronary intervention (PCI) (*n* = 1), increased high-sensitivity C reactive protein (hsCRP) (*n* = 2), increased risk of atrial fibrillation/atrial flutter (AF) (*n* = 4), increased serious bleeding rates (*n* = 1), IPE use in smokers (*n* = 1), people with TG levels between ≥135 mg/dL and 500 mg/dL (*n* = 10), and people with LDL-C levels between >40 mg/dL and ≤100 mg/dL (*n* = 11).

### 3.1. GRADE (Levels of Evidence)

The quality of the evidence was evaluated with the aid of the GRADE system (Grades of Recommendation, Assessment, Development, and Evaluation) [65]. These included the study type, where randomized trials were considered the highest level of evidence, the quality of the trial, and any issues that decreased the quality of the evidence [38]. We employed GRADEpro GDT software from McMaster University in Ontario, Canada, to construct the summary of findings tables. The GRADE assessment revealed that the quality of evidence for IPE’s effects on lipid profiles was generally moderate, with notable concerns related to the risk of bias, inadequate follow-up, losses exceeding 20%, and a high 95% CI (Table 1). The specific GRADE evaluations for each outcome are as follows:

- Total cholesterol: Moderate certainty;
- TG: Moderate certainty;
- HDL-C: Moderate certainty;
- LDL-C: Moderate certainty.

Table S2 within the Supplementary Materials depicts the GRADE analyses.

### 3.2. Quantitative Analysis (Meta-Analysis)

After selecting the relevant studies, the feasibility of performing a meta-analysis was assessed. Where appropriate, lipid profile values were included in the analysis, comparing pre-intervention and post-intervention data. Heterogeneity was quantified using the I^2^ statistic, with values interpreted as follows: 0–40% (minimal heterogeneity), 30–60% (moderate), 50–75% (substantial), and 75–100% (considerable) [39,40]. A *p*-value of less than 0.05 was considered statistically significant for the overall effect size. If studies did not provide the necessary dispersion values, such as SD, CI, or *p*-values, missing SD were estimated. Meta-analysis results were expressed as weighted MD, 95% CI, and *p*-values, and were displayed in forest plots. A random-effects model was used, which reported study heterogeneity, providing more generalizable results [41]. All analyses were conducted using Review Manager software (RevMan 5.4.1).

**Figure 3:**
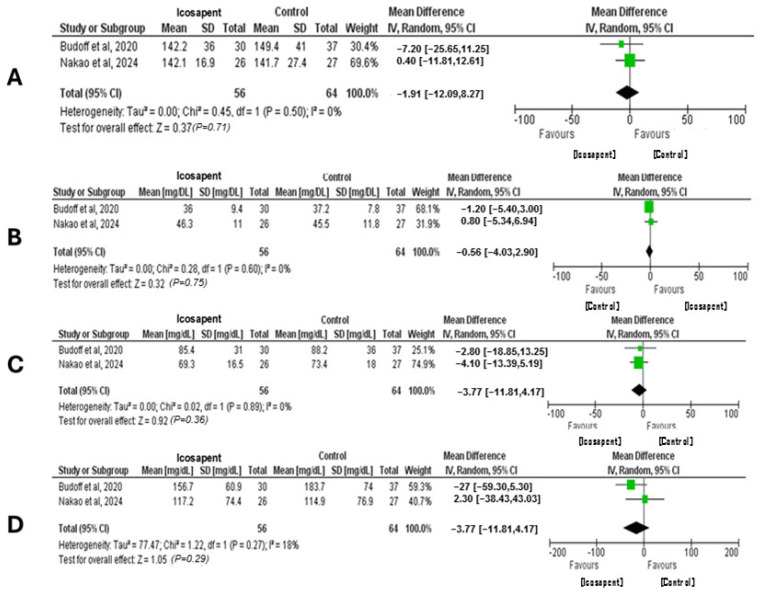
Forest plot assessments for total cholesterol (**A**), HDL-C (**B**), LDL-C (**C**), and TG (**D**). Each plot presents the mean difference (MD) between the intervention and control groups, 95% confidence interval (CI), and corresponding *p*-values [52,64]. The plots indicate no statistically significant changes in lipid parameters following Icosapent Ethyl treatment. The CI crossed the null line for HDL-C, LDL-C, and total cholesterol, indicating non-significant results. Similarly, TG showed a non-significant reduction with minimal heterogeneity (I^2^ = 18%). All analyses were performed using a random-effects model to account for between-study variability. These findings suggest limited overall effects of Icosapent Ethyl on lipid outcomes within the included studies and highlight the need for caution in interpreting pooled estimates based on a small number of trials.

The meta-analysis (Figure 3) evaluated the effects of IPE on several lipid parameters, including HDL-C, LDL-C, total cholesterol, and TG, based on data from two studies. For HDL-C, the meta-analysis included 120 participants from both studies. The results showed no significant change in HDL-C levels (*p* = 0.75), with an MD of −0.56 mg/dL (95% CI: −4.03, 2.9) and no heterogeneity (I^2^ = 0%). In the case of LDL-C, the analysis included 120 participants. The MD was −3.77 mg/dL (95% CI: −11.81, 4.27), indicating no significant effect of IPE on LDL-C levels (*p* = 0.36). Still, the analysis exhibited no heterogeneity (I^2^ = 0%). Two studies with a combined total of 120 participants were analyzed for total cholesterol. The MD was −1.91 mg/dL (95% CI: −12.09, 8.27), indicating no significant impact of IPE on total cholesterol levels (*p* = 0.71). We reported no heterogeneity (I^2^ = 0%). The meta-analysis for TG included 120 participants from two studies. The MD was −15.09 mg/dL (95% CI: −43.29, 13.12), indicating no significant difference in TG levels following IPE treatment (*p* = 0.29). Minimal heterogeneity was detected (I^2^ = 18%).

## 4. Lipid-Lowering Drugs

ASCVD is the leading cause of death worldwide, with hypercholesterolemia and hypertriglyceridemia being its leading causes of progression [66]. Atherogenic plaques are formed due to blood lipoproteins, which permeate the endothelium of blood vessels, promoting an inflammatory process. This culminates in the beginning of atherogenesis, which is a process favored by risk factors such as smoking, high blood pressure, and DM [67]. Therefore, reducing LDL-C levels is the primary goal for mitigating cardiovascular risks [24,68,69].

In the face of ASCVD and subsequent cardiovascular events, LLT was developed, demonstrating efficacy in reducing lipid levels and benefits beyond cardiovascular health [70]. Statins, the main LLT, act in the limiting phase of cholesterol biosynthesis as competitive inhibitors of 3-hydroxy-3-methylglutaryl coenzyme A (HMG-CoA) reductase, preventing its action and thus reducing intrahepatic cholesterol while increasing the hepatic uptake of circulating LDL-C by stimulating the expression of LDL receptors (LDLRs) [71]. Statins promote a reduction in cholesterol levels and the protection of the endothelium through the stimulation of endothelial nitric oxide synthase (eNOS) and the modulation of nitric oxide (NO) synthesis by the serine–threonine protein kinases Akt and caveolin-1, respectively. Furthermore, they promote a reduction in oxidative stress and inflammation by inhibiting the migration and activation of inflammatory cells, reducing the expression of interferon-gamma (IFN-gamma), interleukin-1 (IL-1), tumor necrosis factor (TNF), and endothelial adhesion molecules, as well as inhibiting the production of ROS, delaying atherogenesis [72].

Approximately 3% of the world’s population uses statins to treat dyslipidemia as a primary and secondary prevention for cardiovascular events. However, this drug is highly associated with skeletal muscle toxicity and hepatotoxicity [73]. Factors such as multisystem diseases, chronic renal failure, high doses of statins, alcohol consumption, DM, and obesity constitute risk factors for statin intolerance [74,75,76].

Niemann-Pick C1-Like 1 (NPC1L1) is a transporter protein involved in intestinal fat absorption. PCSK9, a protein produced mainly in the liver, when circulating, binds to LDLRs, favoring their lysosomal degradation, which leads to a reduced hepatic uptake of LDL-C. PCSK9 inhibitors prevent the protein from performing its function, thereby promoting LDL-C reduction. Despite being recommended in the European Atherosclerosis Society guidelines for patients at high cardiovascular risk and/or intolerant to statins, PCSK9 inhibitors are not widely used due to their cost [71,77].

Combination therapy is currently widely prescribed, considering that achieving ideal LDL-C goals with statin monotherapy is challenging in many cases [78]. Combination therapy between statins and ezetimibe or PCSK9 inhibitors was less associated with adverse cardiovascular events than monotherapy with statins, being recommended in cases of intense LDL-C reduction [79].

## 5. Icosapent Ethyl

IPE is a 99.99% pure composition of EPA, whose molecular formula consists of C_22_H_34_O_2_ [1]. EPA is a ω-3 PUFA obtained from fish oil, which chemically consists of a 20-carbon fatty acid containing five double bonds, with the first one located at the third carbon atom from the distal end of the compound tail (C_20_H_30_O_2_) [18]. Currently, there are two approved highly purified formulations of IPE, which contain small proportions of EPA-3 fatty acid and DHA: Vascepa^®^, patented in the US by Amarin Pharma Inc. (Bedminster, New Jersey, USA), and Epadel^®^, patented in Japan by Mochida Pharmaceuticals Co. (Tokyo, Japan) [1,23,80,81].

EPA’s chemical structure is involved in its ability to change cellular membrane properties. The compound can replace arachidonic acid, a 6-carbon fatty acid, in membrane phospholipids, thereby modifying the physical properties of the membrane. This is beneficial due to the reduction of pro-inflammatory and pro-thrombotic factor production from arachidonic acid, such as prostaglandin E2, leukotriene B4, and thromboxane A2, thereby enhancing the levels of anti-inflammatory and anti-thrombotic lipid mediators. However, the ω-3 fatty acid formulations containing DHA can increase LDL-C levels [1,82].

Among the several effects of EPA, the most significant include reduced lipid action, which may reduce steps in the atherogenesis pathway contributing to plaque stabilization, as well as causing antiproliferation, vasodilation, and a reduction in platelet aggregation. Plaque stabilization is related to IPE’s absorption into the phospholipid membrane, stabilizing the formation of coronary plaques and preventing their rupture. Additionally, the compound can lower TG and non-HDL-C levels, improve endothelial function, and prevent cardiovascular events, including MI, coronary revascularization, stroke, and unstable angina [1,83,84].

Furthermore, EPA has been shown to increase eNOS, enhance its coupling efficiency, and improve the NO ratio to peroxynitrite release, promoting better vasodilation and reducing cardiovascular events. Moreover, it has exhibited protective activity against oxidative damage, inhibited monocyte displacement, and converted to macrophages and foam cells [1,83,85,86,87,88,89].

IPE is primarily used in combination with other LLD, typically statins, in patients with established CVD, TG levels ≥ 150 mg/dL, and DM with two or more additional CVD risk factors. This compound also benefits from being associated with therapeutic diets to reduce TG levels ≥ 500 mg/dL [1,82].

IPE is the first fish oil product to have the potential to reduce the risk of ASCVD development in adults, approved by the US Food and Drug Administration [1]. Its indication depends mainly on the guidelines followed. While in Japan, the Japan Atherosclerosis Society asserts that the association of statin treatment and EPA can be helpful in high-risk patients with LDL-C levels ≥ 140 mg/dL, in the US, the National Lipid Association indicates that ω-3 fatty acids should be used as a first-line option for patients with very high TG levels (≥500 mg/dL) and in addition with statins for patients with TG levels between 200 and 499 mg/dL. Furthermore, the Canadian Cardiovascular Society Guidelines stated that EPA is a primary prevention therapy for CVD but also affirmed that formulations containing both EPA and DHA cannot yield the same beneficial results as those containing highly purified IPE [90,91,92].

### 5.1. Icosapent Ethyl and Inflammation

CVD represent a challenge for public health, as they have a progressive prevalence and high financial costs. Among the disorders that this pathology includes, we can mention stroke, coronary artery disease, and heart failure (HF), which have chronic inflammation as an essential factor in their progression [93]. An imbalance in serum lipid levels is associated with the development of CVD, given that LDL-C is considered a pharmacological target for the primary prevention of cardiovascular pathologies. The accumulation of lipids, primarily LDL-C, driven by inflammation, favors atherosclerosis, a highly complex pathophysiological process prevalent in CVD [94].

The inflammatory process is initiated through specific cytokines, known as pro-inflammatory cytokines, which are responsible for developing inflammation. It is worth mentioning the central cytokines that play a role in the establishment of CVD, specifically interleukin-6 (IL-6) and interleukin-1β (IL-1β), which contribute to the progression of the atherosclerotic process by promoting endothelial dysfunction and plaque instability [93]. Furthermore, inflammation, by promoting endothelial dysfunction, facilitates NO reduction, which regulates vascular tone and prevents platelet aggregation, thereby contributing to atherogenesis and vascular injury [87].

IPE has been tested as an option for CVD prevention. According to studies analyzed by Mason et al., IPE was shown to reduce the risk of CVD in patients with high TG through mechanisms such as increasing the rate of beta-oxidation of fatty acids and blocking the action of acyl-CoA:1,2-diacylglycerol acyltransferase (DGAT), which causes a reduction in lipogenesis and VLDL production in the liver, as well as a decrease in serum concentrations of hsCRP and ox-LDL-C. In contrast, it increased the secretion of interleukin-10 (IL-10), a cytokine with anti-inflammatory properties. Such mechanisms favored a reduction in TG, without an increase in the serum concentration of LDL-C, confirming the antiatherogenic function of IPE [95].

Furthermore, EPA metabolism produces E-series resolvins, which are synthesized from RvE1 to RvE4 and 18S-RvE1. Additionally, there are D- and T-series resolvins derived from DHA and docosapentaenoic acid (DPA), respectively, both of which act on the inflammatory response by reducing pro-inflammatory factors and preventing neutrophilic infiltration, a characteristic of inflammation [96]. EPA confers anti-inflammatory effects through competition with arachidonic acid (AA) for the enzymes cyclooxygenase (COX) and lipoxygenase (LOX), responsible for the synthesis of ursoins, especially RvE1 and RvE3, responsible for attenuating inflammation, by reducing the pro-inflammatory signals induced by leukotriene B4 and the migration of polymorphonuclear leukocytes and stimulating the action of macrophages and the production of IL-10, in addition to inhibiting the infiltration of neutrophils, and consequently reducing the production of pro-inflammatory mediators arising from the AA metabolism, thus contributing to cardiovascular benefit [97].

### 5.2. Icosapent Ethyl and Oxidative Stress

Oxidative stress is characterized by an increased production of oxidants in cells and may play a role in atherogenesis. This role is related to the formation of oxidized lipoproteins in the arterial wall and to the potential of ox-LDL-C to form lipid-laden foam cells, which are present in fatty streaks and mature arterial lesions [98]. EPA is beneficial in mitigating inflammatory and oxidative mechanisms related to atherosclerotic plaque development [99]. Its potential in attenuating oxidative stress is primarily associated with its distinct lipophilic and electron-stabilizing properties and its consequent capacity to reduce lipoprotein oxidation [100]. It has been proven that, with its conjugated double bonds along the acyl chain, EPA can scavenge ROS by stabilizing unpaired free radicals through resonance stabilization [101].

Furthermore, studies have shown that EPA can protect HDL-C from oxidation, enhance cholesterol efflux, and improve anti-inflammatory function. EPA’s lipoprotein oxidation inhibition properties can also enhance HDL-C function by inhibiting cytokine-stimulated vascular cell adhesion molecule 1 (VCAM-1) expression and increasing resolvin E3 production. These effects can be attributed to EPA’s molecular three-dimensional structure, which allows it to reside efficiently in cell lipid membranes and lipoprotein particles, where it can interfere with free radical propagation [100].

**Figure 4:**
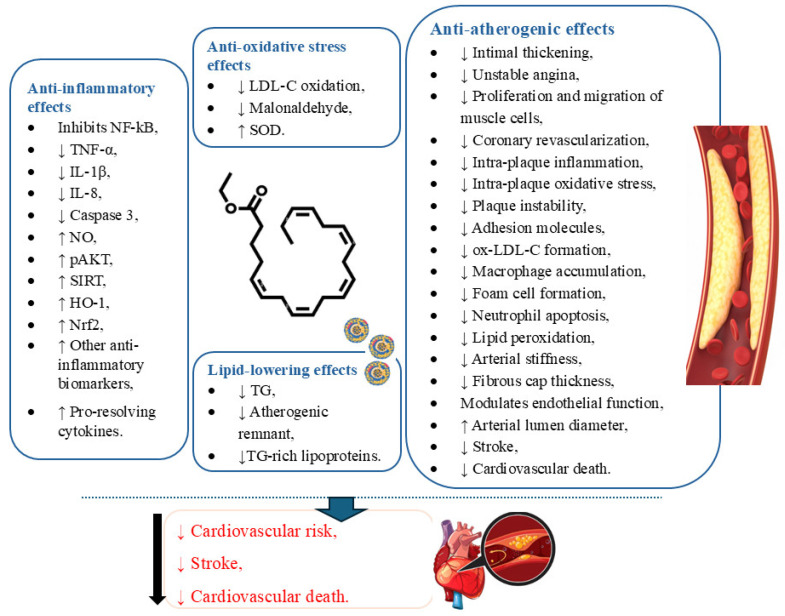
Molecular structure of Icosapent Ethyl and its anti-inflammatory, antioxidant, anti-lipidemic, and anti-atherogenic effects. HO: Heme-oxygenase; IL: Interleukin; LDL-C: Low-density lipoprotein cholesterol; NF-kB: Nuclear factor KB; NO: Nitric oxide; Nrf2: Nuclear factor erythroid 2-related factor 2; ox-LDL-C: Oxidized low-density lipoprotein; pAKT: Phosphorylated-protein kinase B [PKB]; SIRT: Sirtuin; SOD: Superoxide dismutase; TG: Triglycerides; TNF-α: Tumor Necrosis Factor-α.

Moreover, circulating ox-LDL-C can be associated with acute coronary syndrome (ACS), an increased risk of ischemic events, and metabolic diseases [101]. In this scenario, EPA is associated with reduced ox-LDL-C in statin-treated patients with high TG levels, contributing to improved LDL-C clearance [97]. Also, in vitro, EPA showed the potential to inhibit the oxidation of apoB-containing lipoproteins, including LDL-C, small dense LDL-C, and VLDL [99]. In addition, the potent and prolonged antioxidant effects of IPE are also associated with enhanced levels of superoxide dismutase, an enzyme that catalyzes the dismutation of O^2−^ into molecular oxygen and hydrogen peroxide (H_2_O_2_), and decreased levels of malonaldehyde, a biomarker of oxidative damage [99,102]. Figure 4 shows the general effects of IPE.

### 5.3. Clinical Trials Performed with Icosapent Ethyl

Table 1 shows the studies that were included in this review. Table S1 in the Supplementary Materials presents a descriptive table of the biases of the included randomized clinical trials.

Ando et al. [42] evaluated EPA’s potential to prevent the accumulation of atherogenic lipoproteins in the uremic plasma of dialysis patients. During the randomization phase, 22 patients undergoing hemodialysis and 16 undergoing peritoneal dialysis with relatively high plasma levels of lipoproteins and ox-LDL-C were randomized to either the EPA or placebo group. Then, the treatment consisted of administering EPA in an ethyl ester (EE) form (ethyl all-cis-5,8,11,14,17-icosapentanoate) with a purity greater than 91% at a dose of 1800 mg/day for 3 months. After 3 months of treatment, the results showed a 52% reduction in lipoproteins, a 38% reduction in ox-LDL-C, and the normalization of potential lipoprotein abnormalities, as evaluated by gel filtration chromatography. Finally, it is essential to consider the small sample size as a factor that may negatively impact the reliability of the results.

Cawood et al. [43] examined the incorporation of n-3 PUFA into atherosclerotic plaques and their association with plaque inflammation and stability. For 21 days, 121 patients who underwent carotid endarterectomy were randomized to receive either PUFA capsules or placebo capsules of olive oil. After the intervention, some positive aspects were observed, such as those plaques from patients in the PUFA group had fewer foam cells (*p* = 0.0390) than the control group; the EPA content in plaque was inversely associated with plaque instability (*p* = 0.0209), plaque inflammation (*p* = 0.0108), and number of T cells in the plaque (*p* = 0.0097). Moreover, the plaques analyzed showed significantly lower levels of IL-6 (*p* = 0.0395) and intercellular adhesion molecules. Some potential negative points in this study that may have influenced the results include the short duration of treatment and the likely insufficient dose of PUFA.

A study developed by Bays et al. [44], known as the MARINE study, investigated the effects of IPE on lipoprotein particle concentration and size. The intervention consisted of randomizing subjects with TG levels between ≥500 mg/dL and ≤2000 mg/dL in three groups: IPE 4 g/day, IPE 2 g/day, and placebo. Then, after 12 weeks, they measured their lipoprotein particle concentrations and sizes by nuclear magnetic resonance spectroscopy. The results were positive, showing a significant reduction in concentrations of VLDL (−27.9%), LDL-C (−16.3%), small-LDL-C (−25.6%), and HDL-C (−7.4%) and a reduced VLDL particle size (−8.6%), all in the IPE 4 g/day group.

Ballantyne et al. [45] evaluated the safety and efficacy of eicosapentaenoic acid EE (AMR101) in modifying the lipid profile of high-risk statin-treated patients with high TG levels (>200 and <500 mg/dL). Firstly, subjects were randomized into three groups: one receiving AMR101 at 4 g/day, another receiving AMR101 at 2 g/day, and a placebo group. All interventions lasted 12 weeks. Then, the results showed a significant decrease in TG levels in the AMR101 4 and 2 g/day groups by 21.5% and 10.1%, respectively, and in non-HDL-C by 13.6% and 5%, respectively. Moreover, AMR101 4 g/day promoted more significant decreases in TG and non-HDL-C in patients with higher-efficacy statin regimens and more substantial declines in TG in patients with higher baseline TG levels. Furthermore, AMR101 4 g/day decreased LDL-C by 6.2%, apolipoprotein B by 9.3%, total cholesterol by 12%, VLDL by 24.4%, lipoprotein-associated phospholipase A2 by 19%, and hsCRP by 22% compared to placebo. A limitation of this study is that the efficacy of the two doses of AMR101 could not be compared with other currently available therapies, but rather with placebo.

The effect of EPA and DHA on the coronary artery plaque volume associated with statin treatment was evaluated by Alfaddagh et al. [46]. For 30 months, the ω-3 EE group received 1.86 g of EPA and 1.5 g of DHA/day in 4 capsules, while the no ω-3 group (control) received placebo capsules. Then, after the intervention period, coronary plaque volume was assessed using CCTA. The results showed a significant progression of fibrous plaque in the control group, while the ω-3 EE group presented no modifications (5.0% versus −0.1%, respectively; *p* = 0.018). Additionally, in subjects on low-intensity statin therapy, ω-3 EE promoted the attenuation of fibrous plaque progression compared to the control (0.3% versus 4.8%, respectively; *p* = 0.032), while patients on high-intensity statin therapy showed no difference in plaque change between the two treatments. The collected data led to the conclusion that the applied doses of EPA and DHA were beneficial in conjunction with low-statin therapy in preventing the progression of fibrous coronary plaque in patients who adhered to the treatment and maintained controlled LDL-C levels.

A study by Sezai et al. [47] compared the use of EPA versus EPA and DHA in the treatment of hypertriglyceridemia. Over a 3-year period, patients randomized to the EPA group received 1.8 g of IPE three times a day, while those randomized to the EPA + DHA group received 2 g of IPE plus DHA once a day. Six months after the intervention, the results showed that TG, ox-LDL-C, remnant-like particles-cholesterol, and cystatin-C levels were significantly lower in the EPA + DHA group than in the EPA group. A positive aspect to consider is the study’s long-term nature; however, this aspect also led to lower compliance from the subjects regarding medication intake, which may introduce a potential bias. Furthermore, the sample size of 87 subjects may be considerably small.

The study conducted by Miller et al. [48] lasted 12 weeks and involved the randomization of 702 participants with a high cardiovascular risk, TG between 200 and 499 mg/dL, and using statins. Among these, 246 patients were randomized into a subgroup with an hsCRP ≥ 2 mg/L in addition to these characteristics. Patients were randomly assigned to two groups: IPE 4 g/day (*n* = 126) and placebo (*n* = 120). The results demonstrated that patients treated with 4 g/day of IPE, who had an hsCRP ≥ 2 mg/L and were using statins, saw significantly reduced TG levels and other atherogenic parameters without an increase in LDL-C. The study has limitations, including a small sample size and a focus on CVD risks, suggesting that further studies are necessary to assess the impact of IPE on cardiovascular events.

The study by Allaire et al. compared the proportion of individuals whose TG concentrations decreased after administering high doses of DHA and EPA. The randomization included 154 individuals, of whom only 121 completed the research. It was divided into three phases, each lasting 10 weeks, separated by 9-week washouts, and the administration consisted of (1) 2.7 g/day DHA; (2) 2.7 g/day EPA; and (3) 0 g/day DHA + EPA (3 g/day of corn oil). The results demonstrated that DHA resulted in a 45% reduction in serum TG concentrations, while EPA showed a 32% reduction. The trial has some limitations, including that the diet was not evaluated before the study and that participants were instructed to maintain their usual diet. Therefore, it is impossible to exclude the possibility that the essential diet may have influenced the variability in TG and their response to DHA and/or EPA supplementation. Additionally, the results are limited to a population with a high waist circumference and elevated hsCRP levels [49].

Bhatt et al. [50] conducted a study to evaluate the benefits of IPE in patients taking statins with a history of DM and atherosclerosis. The 3146 subjects were randomized into a placebo group and an IPE group, which were followed for an average of 4.9 years. The results obtained in the study point to significant reductions in the IPE group, for example in MI (8.8% to 6.7%; *p* = 0.01), cardiovascular death (6.7% to 4.7%; *p* = 0.007), stroke (4.1% to 2.6%; *p* = 0.02) and all-cause mortality (9.8% to 7.2%; *p* = 0.004). The positive points to be highlighted are the significant sample size and the intervention period, which favor more well-founded results. However, the work also has limitations; among them, the analysis of the groups did not account for the specific characteristics of each group.

The study conducted by Verma et al. [51] utilized the REDUCE-IT samples and aimed to evaluate the effect of IPE in patients with coronary artery revascularization. The study included a sample of 8179 individuals, of whom 1837 were randomly assigned, with a previous coronary artery bypass graft (CABG) history. These were randomized into IPE 4 g/day (*n* = 897) and placebo (*n* = 940). Compared to the placebo group, the results demonstrated a significant reduction in ischemic events in patients with a history of coronary artery revascularization in the IPE group. The study has limitations, as variables such as the time between CABG and randomization and other operative variables were not analyzed.

To analyze whether IPE 4 g/day positively influences a reduction in plaque volume measured by serial MDCT in patients treated with statins, Budoff et al. [52] carried out a study with 80 randomized participants who had coronary atherosclerosis by CCTA, on a stable statin therapy with a low density, in two groups, IPE 4 g/day and placebo. Among the 80 random patients, a loss to follow-up occurred in 15%, resulting in 67 patients who completed the study. This study included assessments of the atherosclerotic plaque at three time points: at the beginning (0 months), at 9 months, and the end of the study (18 months). The results demonstrated that when IPE was added to statin therapy, there was slower plaque progression compared to statin + placebo in the interim analysis (9 months). The study’s short follow-up and small sample size characterize its limitations and do not evaluate long-term results.

“EVAPORATE”, the name given to the ongoing study conducted by Budoff et al., aimed to investigate whether the use of IPE, as a complement to diet and statin therapy, in patients with CVD and imbalanced lipid levels affects the progression of atherosclerotic plaque. The intervention consisted of randomizing individuals with known coronary atherosclerosis and hypertriglyceridemia, as well as stable therapy with statins, diet, and exercise for more than 4 weeks, into two groups, the IPE group, which received 4 g/day, and the placebo group, which were evaluated at the beginning of the study and at intervals of 9 and 18 months. The results demonstrated a significant reduction in the volume of atherosclerotic plaques using IPE. Additionally, treatment with IPE does not favor increased calcium volume in the coronary artery, suggesting a tendency to decrease arterial calcification. However, studies are needed to prove this hypothesis. The study’s limitations include a small sample size and the fact that it does not assess the long-term effects of the intervention [53].

Peterson et al. [54] developed a study to evaluate the effects of IPE on coronary revascularization. Participants who had LDL-C between 41–100 mg/dL, established CVD or diabetes, and used statins for more than 4 weeks were randomized into the IPE 4 g/day and placebo groups. The results demonstrated a significant reduction in revascularization events in patients undergoing IPE treatment. The study’s limitations are exemplified by the *p*-value results, which do not confirm the researcher’s hypotheses. However, a positive aspect of the work is the relatively large sample size and the duration of the intervention that patients underwent.

Furthermore, Peterson et al. [55] conducted another study in 2022 to evaluate the effects of IPE on reducing ischemic events in patients undergoing PCI. The study included a group of 8179 randomized by REDUCE-IT. within this group, the 3408 patients who underwent PCI were distributed into the IPE 4 g/day and placebo groups for analysis. The analysis demonstrated that IPE substantially reduced ischemic events in patients with PCI. The degree of risk for ischemic events is more significant the closer to the PCI period; therefore, some limitations of the study can be cited, such as that the patient’s PCI time was not considered, so it is not possible to state whether the effects observed may vary according to the degree of risk of ischemic events.

Gaba et al. [56] conducted a study to evaluate the impact of IPE on ischemic events in patients with a history of MI. The study used the REDUCE-IT sample, comprising 3693 patients randomly assigned to the IPE 4 g/day and placebo groups, who were followed for 4.8 years. The results obtained by Gaba et al. demonstrated a significant reduction from 26.1% to 20.2% in ischemic events in patients with a previous MI. The study data are pre-established and not specific to the subgroup analyzed.

The study conducted by Maki et al. [57] randomly assigned 100 individuals, aged 60.3 years on average, with TG ranging from 150 to 499 mg/dL, to two groups: EPA + DPA-free fatty acid (FFA) and EPA-EE. The participants were then subjected to two 28-day treatment periods, separated by a 28-day interval. The results obtained by the study demonstrated that basal TG levels were reduced from 20.9% to 18.3% without increasing LDL-C levels. The study has several strengths, including a relatively large sample size and a study design that measured blood levels twice daily.

Selvaraj et al. [58] conducted a study using data from REDUCE-IT, in which 8179 individuals were randomized. Among these, 1446 with a history of HF were randomly assigned to two groups: IPE (*n* = 703) and placebo (*n* = 743). The results showed similar reductions in cardiovascular risk for patients with and without HF. The study’s limitations are related to the lack of ejection fraction data to determine the benefit consistency when applied to HF patients with a reduced or preserved ejection fraction and information on the cause of HF. The strengths include the sample size, which comprised nearly 1500 individuals, and the follow-up time, which lasted an average of 4.6 years.

A study developed by Olshansky et al. [59] analyzed the increased cases of AF hospitalization that developed in the REDUCE-IT trial. The REDUCE-IT intervention consisted of randomizing 8179 subjects into a placebo group (*n* = 4000) and an IPE group (*n* = 4000), who received 4 g/day of IPE (2 g twice daily with meals) for 4.9 years. The results related to AF hospitalization cases in these patients showed that the event rates were higher in patients with prior AF (12.5% versus 6.3%, IPE versus placebo; *p* = 0.007) compared to those without prior AF (2.2% versus 1.6%, IPE versus placebo; *p* = 0.09). Furthermore, serious bleeding rates were higher in patients with (7.3% versus 6.0%, IPE versus placebo; *p* = 0.59) than those without prior AF (2.3% versus 1.7%, IPE versus placebo; *p* = 0.08). It is essential to consider that the large sample size and extended intervention time contribute to the reliability of the results. In contrast, some limitations include the fact that “REDUCE-IT” was not designed to evaluate the incidence of AF events and the antiarrhythmic or proarrhythmic effects of IPE in that population. Additionally, the group that suffered from AF events was small, and the mechanism relating the IPE to these events remains unknown.

Miller et al. [60] conducted a study that involved a post hoc analysis examining the relationship between smoking history and IPE use in the REDUCE-IT trials. The subjects were divided into three groups: current smokers (*n* = 1241), former smokers (*n* = 3672), and never smokers (*n* = 3264). The effects of IPE use were evaluated in all groups. The results showed that IPE use in current and former smokers was associated with significant reductions in time to the following events: cardiovascular death, non-fatal MI, non-fatal stroke, coronary revascularization, or hospitalization for unstable angina (*p* < 0.0001) and in total events (*p* < 0.0001). There were also identified reductions in cardiovascular death as well as non-fatal MI or stroke (*p* < 0.0001) in both the current and former smokers’ groups. The estimated rates of first occurrences of cardiovascular death, non-fatal MI, non-fatal stroke, coronary revascularization, or hospitalization for unstable angina in current smokers were 23.8% and in former smokers 23.0%, in contrast to never smokers on placebo (25.7%). Some favorable aspects of this trial were the large sample size and the prolonged intervention period. Meanwhile, some limitations include that the groups were defined based on self-report, and the number of cigarettes smoked daily, the number of pack-years, or the duration of time since former smokers quit were not collected.

A clinical trial conducted in China aimed to evaluate the efficacy of IPE in treating high TG levels. The intervention took place over 12 weeks in 373 patients randomized to an IPE 4 g/day group, an IPE 2 g/day group, and a placebo group. The results showed a reduction in blood TG levels of 28.4%, 12.0%, and 6.2% in these groups, respectively. Additionally, IPE at a dose of 4 g/day reduced total cholesterol levels by 19.9% compared to baseline (*p* < 0.001) and at a dosage of 2 g/day led to a 5.0% reduction in TG levels (*p* = 0.361). The study presented some positive points, such as that other lipoproteins associated with TG were significantly reduced by IPE 4 g/day, and all the study groups tolerated the medication well. However, there were limitations, such as the short study time, so the medication’s long-term effects and its impacts on cardiovascular risks were not evaluated [61].

The effects of IPE use on patients with recent (<12 months) ACS were evaluated in a post hoc analysis of the REDUCE-IT trial. The group of recent ACS consisted of 840 patients. The results showed that IPE reduced the incidence of the first primary composite outcome, which consisted of cardiovascular death, non-fatal MI, non-fatal stroke, coronary revascularization, or hospitalization for unstable angina, by 37% (*p* = 0.002), and total primary composite outcomes by 36% (*p* = 0.01). Also, the absolute risk reduction in the first primary outcome with IPE was 9.3%. Moreover, IPE reduced the incidence of the first key secondary composite outcome, which consisted of cardiovascular death, non-fatal MI, or nonfatal stroke, by 36% (*p* = 0.01) and total key secondary composite outcomes by 28% (*p* = 0.12). Furthermore, IPE lowered cardiovascular death and non-fatal MI by 36% (*p* = 0.03). It lowered urgent or emergent revascularization by 44% (*p* = 0.009). Considering recent ACS patients on dual antiplatelet therapy, the percentage with at least one treatment-emergent bleeding adverse event was 7.7% in the IPE treatment group and 9.4% in the placebo (*p* = 0.46). The large sample size and the length of intervention were strengths of this trial [62].

Szarek et al. [63] evaluated in this post hoc analysis of the REDUCE-IT trial the cardiovascular benefit of IPE associated with different lipoprotein(a) (Lp(a)) levels. Of all the randomized subjects, only 86% had baseline Lp(a) assessments, with a median concentration of 11.6 mg/dL. The results showed that Lp(a) was significantly related to both first and total major adverse cardiovascular events (*p* < 0.0001) and significantly reduced the incidence of first major adverse cardiovascular events in subgroups with concentrations of ≥50 or <50 mg/dL. It is essential to consider that the large sample size and extended intervention time contribute to the reliability of the results.

The study developed by Nakao et al. [64] evaluated the effects of EPA/DHA on atherosclerotic plaques, as measured by cardiac magnetic resonance, in patients using statins and with coronary artery disease after 12 months. The intervention consisted of randomizing patients into three groups: a 2 g/day EPA/DHA group, a 4 g/day EPA/DHA group, or a no-treatment group. The results demonstrated a reduction in the plaque-to-myocardium signal intensity ratio of −0.15 in the 2 g/day and 4 g/day EPA/DHA groups. At the same time, no changes were observed in the untreated group. The study has several limitations, including a relatively small sample size, the absence of a blinded study design, and an unknown duration of follow-up, which may be insufficient to fully evaluate the effect of EPA/DHA on atherosclerotic plaque.

Bakbak et al. [27] evaluated the potential of IPE to regulate vascular regenerative cell content in individuals with mild to moderate hypertriglyceridemia. A sample of 70 people was randomized into two groups, receiving either the intervention of IPE (4 g/day) or their usual care for 3 months. The results demonstrated the increased mean frequency of high aldehyde dehydrogenase activity (ALDHhi) side scatter low CD133+ cells (*p* = 0.02), decreased overall ALDHhi side scatter low cell frequency, reduced oxidative stress, and increased ALDHhi side scatter granulocyte precursor cell content in the IPE group, proving that IPE can interfere in vascular regenerative cell content, promoting cardioprotective effects. Meanwhile, as a limitation, the article presents a small sample size, lacks a placebo comparison group, and has a short intervention period.

#### Interpretations of the Meta-Analysis from the Included Clinical Trials Performed with Icosapent Ethyl Assessment

Nevertheless, our quantitative analysis failed to detect a significant positive effect of IPE on the lipid profile. The statistical analyses used by the included studies enabled us to conduct a meta-analysis for total cholesterol, LDL-C, HDL-C, and TG [52,64].

Regarding total cholesterol, we reported a mean SD of 1.91 (95% CI: −12.09, 8.27) lower than that of the control group after the intervention period (*p* = 0.71). Regarding HDL-C, we noted a mean SD of 0.56 (95% CI: −4.03, 2.9), which was lower than that of the control group after the treatment period (*p* = 0.75). When investigating LDL-C, we observed a mean SD of 3.77 (95% CI: −11.81, 4.27) lower than the control group after the intervention period (*p* = 0.36). Concerning TG, we observed a mean of 15.09 SD (95% CI: −43.29, 13.12) lower than the control group after the exercise intervention period (*p* = 0.29).

According to the results, we do not support the notion that IPE significantly affects lipid profiles. Nevertheless, this finding contradicted our initial hypothesis since a recent meta-analysis reported the impact of ω-3 PUFA in type 2 DM [103]. The authors evaluated a total of 46 randomized controlled trials (4991 participants) and found that ω-3 PUFA supplementation improved HDL-C (0.05; 95% CI: 0.02 to 0.08) levels, TG (−0.36; 95% CI: −0.48 to −0.25), and total cholesterol (−0.22; 95% CI: −0.32 to −0.11).

We should exercise caution in this interpretation. Our meta-analysis included only two references [52,64] because the remaining four references [44,45,46,48] did not provide mean, SD, and standard errors, which prevented us from conducting a meta-analysis. Additionally, the remaining publications not included in the meta-analysis did not present lipid profile values, as they focused on different parameters.

Therefore, the evidence-level analysis, as assessed by GRADE, showed a low certainty.

### 5.4. Limitations and Future Research Endeavors Based on the Included Studies

Based on this systematic review, IPE effectively mitigated CVD and outcomes while lowering blood lipids among the studied populations. The intervention also imposed limited and no severe side effects. However, when applying statistical methods and performing a meta-analysis of the included results, it is worth noting that the evidence on IPE is limited, and the intervention may not adequately protect against ASCVD and their outcomes. This limitation stems from several critical factors not fully explored in the current analysis. These include variations in study design, differences in dosages of IPE, heterogeneity in patient populations (such as baseline cardiovascular risk, age, gender, and comorbidities), and the duration of interventions. Furthermore, the small number of eligible studies and their relatively modest sample sizes reduce the statistical power of the meta-analysis and limit the generalizability of the findings. These limitations highlight the need to interpret the findings of this meta-analysis with caution. Several potential sources of heterogeneity have contributed to the non-significant results observed in lipid outcomes. First, inconsistencies in intervention protocols—such as differences in IPE dosages, the durations of follow-up, and whether participants were concurrently using statins—likely affected the comparability across studies. Additionally, variations in patient demographics, including age, sex distribution, baseline lipid levels, comorbidities (e.g., diabetes, hypertension), and cardiovascular risk profiles, have influenced individual study outcomes and diluted the overall effect size. Second, the limited number of eligible trials, with small sample sizes and short follow-up periods in some studies, restricted the statistical power and increased the risk of type II errors. Furthermore, not all studies reported complete data or standardized outcome measures, making it difficult to conduct robust sensitivity analyses or subgroup assessments. Future research should prioritize more extensive, well-controlled trials with standardized reporting and longer follow-up durations to better assess IPE’s long-term effects and safety profile. Moreover, individual participant data meta-analyses may help elucidate the role of specific patient characteristics and treatment variables in modulating the efficacy of individual participant data. Nevertheless, IPE possesses anti-inflammatory, anti-thrombotic, and anti-atherogenic properties, highly related to cardiovascular protection. Therefore, future research endeavors must fully assess IPE as an anti-lipid therapy and cardiovascular protector.

In this scenario, mechanistic studies are crucial in elucidating the mechanisms by which IPE exerts its cardioprotective effects, particularly in diabetes and other metabolic disorders. Genetic and epigenetic research are also areas of interest. Nutrigenomics studies the impacts of food and dietary components on the gene expression of genetic variants and other nutritional factors. This field of study focuses on how nutrients and functional foods interact with the genome at the molecular level, allowing insights into how food compounds or dietary constituents may influence human health [104]. Future research should investigate the impact of IPE on metabolic health at the genetic level, exploring how genetic variations influence responses to IPE supplementation. Genetic variations may affect individual dietary intake and supplement responses [105]. Ultimately, research must investigate the rationalization of integrating IPE with nanomedicine or nanoparticles. Nutritional supplements may present poor stability and absorption in various conditions [106]. Combined with nano-delivery systems and nanotechnology, IPE can enhance its cardioprotective effects, promote more nuanced anti-inflammatory and antioxidant properties, and provide targeted cardiovascular protection.

Alongside the future research endeavors mentioned above, it is crucial to research the differing effects of IPE among different populations. Proposing studies on the impact of IPE in other age groups, ethnic groups, or individuals with different or cumulative comorbidities should be sufficient to emphasize how genetic, environmental, and lifestyle factors may influence the drug’s efficacy across a diverse patient population. Additionally, especially for the elderly with cumulative comorbidities, including diabetes, hypertension, and complications, examining the effects of IPE in long-term studies is essential for understanding the drug’s sustained benefits and its potential adverse effects over an extended period. In these groups, examining the impact of IPE in combination with other ω-3 supplements may also be an essential avenue for research to understand its relative efficacy, considering complementary therapies with other compounds.

Besides its benefits on cardiovascular health, IPE is often considered cost-effective for primary and secondary cardiovascular prevention. Studies comparing its effectiveness to other LLD, including statins and PCSK9 inhibitors, have demonstrated that, at an annual price, IPE provides cardiovascular protection at a lower cost. In Germany, Michaeli et al. [107] found that IPE is more cost-effective for cardiovascular protection than PCSK9 inhibitors, with an annual price of GBP 2064 in the UK, which is significantly lower than that of PCSK9 therapy. Another study conducted by Weintraub et al. [108] in the United States demonstrated that IPE therapy for lipid-lowering effects costs USD 4.59 per day, offering better outcomes than standard lipid-lowering care, which costs USD 11.48 per day. In Canada, Lachaine et al. [109] evaluated IPE’s cost-effectiveness in reducing ischemic cardiovascular events among the Canadian population. Their results affirmed that IPE reduces ischemic cardiovascular events in statin-treated patients with elevated TG by an incremental cost of USD 12,523, representing an estimated 0.29 more quality-adjusted life years (QALYs). On the other hand, this additional 0.29 years corresponds to an incremental cost-effectiveness ratio of USD 42,797/QALY gained, with a probability of 70.4% to 98.8% that IPE is more cost-effective than placebo in statin-treated patients. Finally, in another study, Weintraub et al. [110] evaluated the cost-effectiveness of IPE in patients with hypertriglyceridemia who were already receiving statin treatment. Their results demonstrated that IPE offered better cardiovascular outcomes than standard care with statins at USD 22,311/QALY, ultimately leading to a 58.4% lifetime probability of being cost-effective and providing better cardiovascular protective effects.

## 6. Conclusions

Based on our systematic review, IPE might be considered significant in effectively lowering TG levels and reducing cardiovascular death, non-fatal MI, non-fatal stroke, coronary revascularization, or hospitalization for unstable angina. However, it is worth noting that when applying statistical methods to the literature review, we found that IPE does not sufficiently protect against ASCVD. Nevertheless, it is worth noting that the evidence was mainly based on patients undergoing statin therapy. Additionally, it is essential to note that IPE exhibits multiple effects that are believed to enhance cardiovascular health and reduce cardiovascular outcomes, including anti-inflammatory, anti-thrombotic, and anti-atherogenic properties. In contrast, its adverse effects include, mainly, AF and an increased risk of bleeding. Therefore, IPE may be considered a promising therapy against ASCVD in association with other LLD, especially statins, for patients with very high TG levels. Although quantitative evidence does not confirm the benefits of IPE, only two studies were included. Moreover, even if not significant, it is known that a reduction in TG reduces cardiovascular outcomes, especially in those suffering from diabetes and other metabolic complications [111].

## Figures and Tables

**Table 1:** Studies show the effects of Icosapent Ethyl on cardiovascular disease risks.

Ref.	Model/Country	Population	Intervention/Comparison	Outcomes	Side Effects
[42]	Clinical study, 7-month/Japan.	In dialysis treatment, 38 subjects, 5♀, 33♂, 38–65 y.	The patients were randomly allocated to either the control group or the treatment group, which received a highly purified EPA in EE form (ethyl all-cis-5,8,11,14,17-icosapentanoate) at 1800 mg/day. The study was conducted in the baseline observation, treatment (3 months), and washout period (3 months).	Treatment with EPA significantly reduced the levels of both remnant lipoproteins and ox-LDL-C by 52% and 38%, respectively. Furthermore, the gel filtration chromatography of lipoproteins showed that the treatment also normalized other potential abnormalities in lipoproteins.	One patient reported mild headache and diarrhea, but these symptoms soon disappeared without any treatment.
[43]	Randomized, double-blind, placebo-controlled/United Kingdom.	121 subjects, 68♂, 32♀,41–91 y, were destined to undergo carotid endarterectomy.	Sixty-one subjects were randomized to receive control capsules (olive oil), and the other sixty subjects received n-3 PUFA EE capsules twice daily for 21 days until surgery.	Plaques from patients in the *n*-3 PUFA group contained fewer foam cells than those in the control group. EPA content in plaque was inversely associated with plaque instability, inflammation, and the number of T cells; plaques showed significantly lower levels of IL-6 and intercellular adhesion molecule.	No side effects were reported.
[44]	Phase three, multi-center, placebo-controlled, randomized, double-blind, 12-week study/United States of America.	177 subjects, 133♂, 44♀, with very high TG levels (≥500 mg/dL and ≤2000 mg/dL).	Subjects were randomized into three groups: IPE 4 g/day, IPE 2 g/day, and placebo. After 12 weeks, nuclear magnetic resonance spectroscopy measured lipoprotein particle concentrations and sizes.	The IPE 4 g/day group showed a significant reduction in concentrations of VLDL (−27.9%), total LDL-C (−16.3%), small LDL-C (−25.6%), and total HDL-C (−7.4%), as well as a reduction in VLDL particle size (−8.6%).	No side effects were reported.
[45]	Phase three, multi-center, placebo-controlled, randomized, double-blinded, 12-week clinical trial/United States of America.	702 subjects, 431♂, 271♀, >18 y in high-risk statin-treated patients with high TG levels (>200 and <500 mg/dL).	Subjects were randomized to 3 groups: one receiving AMR101 (ω-3 fatty acid agent containing > 96% pure IPE) 4 g/day for 12 weeks, another receiving AMR101 2 g/day for 12 weeks, or a placebo group.	AMR101 4 and 2 g/day significantly decreased TG levels by 21.5% and 10.1%, respectively, and non-HDL-C by 13.6% and 5%, respectively. Additionally, AMR101 4 g/day promoted more significant decreases in TG and non-HDL-C in patients with higher-efficacy statin regimens and more substantial reductions in TG in patients with higher baseline TG levels.	The reported side effects included diarrhea, nausea, nasopharyngitis, arthralgia, and belching.
[46]	Randomized, controlled, parallel, 30-month clinical trial/United States of America.	285 subjects, 21–80 y, and with stable coronary artery disease on statins.	Subjects were randomized into ω-3 EE (1.86 g of EPA and 1.5 g of DHA per day, *n* = 143) or the no ω-3 group (control, *n* = 142) for 30 months.	Controls showed significant progression of fibrous plaque, whereas the ω-3 EE group presented no modifications. Among the subjects on low-intensity statin therapy, ω-3 EE promoted the attenuation of fibrous plaque progression. Patients on high-intensity statin therapy showed no changes in plaque in either treatment group.	No serious musculoskeletal events in the ω-3 EE group compared with the control.
[47]	Randomized, single-blinded, 3-year clinical trial/Japan.	87 subjects, 59♂, 28♀, 58–80 y with untreated hypertriglyceridemia (TG level ≥ 150 mg/dL) and who had undergone cardiac surgery.	Subjects were randomized to the EPA group, which received 1.8 g of IPE 3×/day, or the EPA + DHA group, which received 2 g of IPE + DHA once daily for 3 years.	Six months after completing the intervention, the results showed that TG, remnant-like particles cholesterol, ox-LDL-C, and cystatin-C levels were significantly lower in the EPA + DHA group than in the EPA group.	In the EPA group, *n* = 2 reported arrhythmia, and *n* = 1 HF; in the EPA + DHA group, 1 patient reported HF.
[48]	Phase three, randomized, double-blind, placebo-controlled clinical study/United States of America.	702 participants, 136♂, 110♀ with a history of high CV risk, TG 200–499 mg/dL, LDL-C 40–99 mg/dL controlled on statin therapy and elevated hsCRP (hsCRP ≥ 2 mg/L).	Participants were randomized to IPE 4 g/day (*n* = 126) and placebo (*n* = 120).	IPE 4 g/day significantly reduced fasting TG by 19.9% (*p* < 0.0001). Apolipoprotein B, apolipoprotein C-III, and markers of oxidation and inflammation, including hsCRP, ox-LDL-C, and Lp-PLA2, were significantly decreased with IPE 4 g/day compared to placebo (*p* = 0.0213 to <0.0001).	Nausea, diarrhea, nasopharyngitis, and arthralgia.
[49]	Double-blind, controlled, crossover, randomized study/Canada.	154 subjects, 85♀, 36♂, 36–68 y, with WC ≥ 80 cm for ♀, ≥94 cm for ♂ and serum hsCRP concentrations > 1 and <10 mg/L.	The study was divided into 3 phases, each lasting 10 weeks, separated by 9-week washouts. In total, 3 × 1 g of 90% purified long-chain *n*-3 PUFA capsule/day was provided throughout the 3 phases: (1) 2.7 g/day DHA; (2) 2.7 g/day EPA; (3) 0 g/day DHA + EPA (3 g/day of corn oil). The objective was to compare the effect of DHA and EPA on the plasma concentrations of hsCRP.	The overall mean reduction in serum TG concentrations was more significant with DHA than with EPA (45 and 32%, respectively; *p* < 0.001).	No side effects were reported.
[50]	Phase three B, international, multi-center, prospective, randomized, double-blinded, placebo-controlled, parallel-group trial/United States of America.	3146 subjects, 1015♀, 2131♂, 59–71 y statin-treated patients with TG ≥ 135 and <500 mg/dL, LDL-C > 40 and ≤100 mg/dL, and a history of atherosclerosis or DM with additional CV risk factors.	Patients were randomized into the placebo group (*n* = 1598) and the IPE group (*n* = 1548), receiving 4 g/day of IPE. Subjects were followed for an average of 4.9 years.	Results showed that all the following factors were significantly reduced in the IPE group: CV death (6.7% to 4.7%; *p* = 0.007), MI (8.8% to 6.7%; *p* = 0.01), stroke (4.1% to 2.6%; *p* = 0.02), and all-cause mortality (9.8% to 7.2% *p* = 0.004).	Diarrhea, constipation, dysphagia, belching, AF, and bleeding are treatment-emergent adverse events of any type.
[51]	Phase three B, double-blind study/United States of America (leading country).	8179 patients, with a stable dose of statin for at least 4 weeks at baseline, LDL-C between 41 mg/dL and 100 mg/dL, and TG between 135 mg/dL and 500 mg/dL.	Among the 8179 randomized patients, those who underwent coronary artery revascularization were selected and randomly assigned to receive either IPE 4 g/day (*n* = 897) or placebo (*n* = 940).	There was a significant reduction in the primary outcome; the HR was 0.76, with a *p*-value of 0.004. In the secondary outcome, HR = 0.69 and *p* = 0.001. In the first outcome, plus subsequent or recurrent ischemic events, the HR was 0.64 with *p* = 0.0002, compared to placebo.	Bleeding or AF requiring hospitalization for at least 24 h are the main adverse effects.
[52]	Prospective, placebo-controlled, randomized, double-blind study/United States of America.	80 subjects, 36♂, 31♀, 51–63 y, with coronary atherosclerosis by CCTA (≥1 angiographic stenosis with ≥20% narrowing), on stable statin therapy with LDL-C levels of 40–115 mg/dL and persistently high levels of TG (135–499 mg/dL).	Patients were randomized 1:1 into the IPE 4 g/day (*n* = 30) and placebo groups (*n* = 37); additionally, they underwent an MDCT examination to assess plaque volume in CCTA at 0, 9, and 18 months.	At 9 months, there was no significant change in LAP between groups (*p* = 0.469). There was a slowdown in total non-calcified plaque (sum of LAP, fibrofatty, and fibrous plaque) (*p* = 0.010), total plaque (non-calcified + calcified plaque) (15% vs. 26%, *p* = 0.0004), fibrous plaque (17% vs. 40%, *p* = 0.011), and calcified plaque (−1% vs. 9%, *p* = 0.001).	The article reported no adverse effects.
[53]	Multi-center randomized, double-blind, placebo-controlled trial/United States of America.	Eighty subjects, 31♀, 37♂, 51–63 y, with known coronary atherosclerosis, elevated fasting TG levels (135–499 mg/dL), and LDL-C levels between ≥40 and ≤115 mg/dL, as well as on stable statin therapy.	Participants were divided into an IPE or a placebo group to evaluate the effect of IPE 4 g/day on coronary plaque progression, as determined by CCTA, compared to an oil placebo mineral. Patients underwent three MDCT exams: the first at the beginning of the study, an intermediate one at 9 months, and a final one at 18 months.	Significant reductions in LAP in the IPE group. Total plaque (−9% with IPE vs. +11% with placebo, *p* = 0.002), total non-calcified plaque (−19% vs. +9%, *p* = 0.0005), fibrofatty (−34% vs. +32%, *p* = 0.0002), fibrous (−20% vs. 1%, *p* = 0.003), and calcified plaque (−1% vs. +15%, *p* = 0.053.	No side effects were reported.
[54]	Phase three B, double-blind, randomized, placebo-controlled trial/United States of America (leading country).	8179 patients, with a stable statin dose for at least 4 weeks at baseline, LDL-C between 41 mg/dL and 100 mg/dL, and TG between 135 mg/dL and 499 mg/dL.	Patients were randomized into two groups: IPE 4 g/day (*n* = 4000) and placebo (*n* = 4000), and underwent the intervention for an average of 4.8 years.	IPE significantly reduced the need for PCI, with an HR of 0.68 and *p* < 0.0001, and the need for CABG surgery, with an HR of 0.61 and *p* = 0.0005.	Minor bleeding events, with a tendency toward increased significant bleeding, and a slightly increased risk of AF.
[55]	Randomized double-blind, multi-center, placebo-controlled trial/United States of America (leading country).	8179 patients, with a stable dose of statin for at least 4 weeks at baseline, LDL-C between 41 mg/dL and 100 mg/dL, and TG between 135 mg/dL and 499 mg/dL.	Patients randomized by REDUCE-IT who underwent PCI were distributed into the IPE 4 g/day and placebo groups, followed for a median of 4.8 years.	34% reduction in the primary composite outcome and a 34% reduction in the primary secondary composite outcome of CV death, non-fatal MI, or non-fatal stroke, presenting HR = 0.66.	Documented AF that required emergency treatment or hospitalization.
[56]	Randomized double-blind, multi-center, placebo-controlled trial/United States of America (leading country).	8179 patients, with a stable dose of statin for at least 4 weeks at baseline, LDL-C between 41 mg/dL and 100 mg/dL, and TG between 135 mg/dL and 499 mg/dL.	Among those randomized, 3693 patients with a history of previous MI were assigned to either IPE 4 g/day (*n* = 1870) or placebo (*n* = 1823) groups, followed for an average of 4.8 years.	Significant reduction in the incidence of CV death, MI, stroke, coronary revascularization, or hospitalization for unstable angina from 26.1% to 20.2% compared to placebo (HR: 0.74; *p* = 0.00001). The secondary outcome (CV death, MI, or stroke) was reduced from 18.0% to 13.3% (HR: 0.71; *p* = 0.00006).	The article reported no adverse effects.
[57]	Open, randomized, 2-way crossover clinical trial/United States of America.	100 subjects, 43♂, 57♀, mean 60.3 y, with fasting TG of 150–499 mg/dL.	The trial consisted of two 28-day treatment periods, separated by an interval of at least 28 days. Subjects were randomized to EPA-EE (first period) and EPA + DPA-FFA (second period) or vice versa, taking two capsules twice daily with meals.	EPA-EE increased hsCRP by 8.5% (*p* = 0.034). EPA + DPA-FFA increased DHA by 1.7%; EPA-EE decreased DHA by 3.3% (*p* = 0.011).	Subjects receiving EPA + DPA-FFA reported nausea, diarrhea, belching, and arthralgia. Subjects receiving EPA-EE reported diarrhea, arthralgia, and constipation.
[58]	Randomized double-blind, multi-center, placebo-controlled trial/United States of America (leading country).	8179 patients, with a stable dose of statin for at least 4 weeks at baseline, LDL-C between 41 mg/dL and 100 mg/dL, and TG between 135 mg/dL and 499 mg/dL.	Patients were randomized into IPE (*n* = 703) and placebo (*n* = 743).	In patients with HF, IPE reduced TG (33.5 mg/dL, or 15.4% *p* < 0.0001) and hsCRP (35.1% *p* < 0.0001) from baseline up to 2 years compared to placebo.	The article reported no adverse effects.
[59]	Phase three B, international, multi-center, prospective, randomized, double-blinded, placebo-controlled, parallel-group trial/United States of America (leading country).	8179 subjects, 59–71 y statin-treated patients with TG levels between ≥135 and <499 mg/dL, LDL-C > 41 and ≤100 mg/dL, and a history of atherosclerosis or DM with additional CV risk factors.	Patients were randomized into the placebo group (*n* = 4000) and the IPE group (*n* = 4000), which received 4 g/day of IPE (2 g twice daily with meals). Subjects were followed for an average of 4.9 years.	AF hospitalization event rates were higher in patients with prior AF (12.5% versus 6.3%, IPE versus placebo; *p* = 0.007) than those without prior AF (2.2% versus 1.6%, IPE versus placebo; *p* = 0.09).	Diarrhea, constipation, dysphagia, belching, AF, and bleeding are treatment-emergent adverse events of any type.
[60]	Phase three B, international, multi-center, prospective, randomized, double-blinded, placebo-controlled, parallel-group trial/United States of America (leading country).	8179 subjects, 59–71 y statin-treated patients with TG levels between ≥135 and <499 mg/dL, LDL-C > 41 and ≤100 mg/dL, and a history of atherosclerosis or DM with additional CV risk factors.	Patients were randomized into two groups: the placebo group (*n* = 4000) and the IPE group (*n* = 4000), which received 4 g/day of IPE (2 g twice daily with meals). Subjects were followed for an average of 4.9 years. Then, in the post hoc analysis, the subjects were classified into three groups: current smokers (*n* = 1241), former smokers (*n* = 3672), and never smokers (*n* = 3264).	Significant reductions in time to CV death, non-fatal MI, non-fatal stroke, coronary revascularization, or hospitalization for unstable angina (*p* < 0.0001) and in total events (*p* < 0.0001) were observed in current and former smokers who used IPE. The estimated rates of first occurrences were 23.0% in former smokers, in contrast to 25.7% in never smokers on placebo. Also, there were reductions in CV death as well as non-fatal MI or stroke (*p* < 0.0001) in both current and former smoker groups.	Diarrhea, constipation, dysphagia, belching, AF, and bleeding are treatment-emergent adverse events of any type.
[61]	Randomized, double-blind, multi-center, placebo-controlled clinical trial, phase three/China.	373 subjects, 93♀, 280♂, median 48.9 y, with reduced TG levels by 5.6–22.6 mmol/L with stable diet and physical activity.	Subjects were randomized into three groups: (1) IPE 2 g/day: one IPE, 1 g and one placebo capsule 2×/day; (2) IPE 4 g/day group, two IPE capsules, each with a dosage of 1 g 2×/day; and placebo group, two placebo capsules 2×/day/6–8 weeks, and if mean TG levels were 5.6–22.6 mmol/L after screening, these participants were selected for treatment phase/12 weeks.	There was a reduced TG level by 28.4%, 12.0%, and 6.2% in the groups receiving placebo, IPE 2 g/day, and IPE 4 g/day, respectively. IPE at a dose of 4 g/day reduced total cholesterol levels by 19.9% compared to baseline (*p* < 0.001), and IPE at a dosage of 2 g/day led to a reduction in TG level of 5.0% (*p* = 0.361).	Diarrhea and urticaria.
[62]	Phase three B, international, multi-center, prospective, randomized, double-blinded, placebo-controlled, parallel-group trial/United States of America (leading country).	8179 subjects, 59–71 y statin-treated patients with TG levels between ≥135 and <499 mg/dL, LDL-C > 41 and ≤100 mg/dL, and a history of atherosclerosis or DM with additional CV risk factors.	Patients were randomized into two groups: the placebo group (*n* = 4000) and the IPE group (*n* = 4000), which received 4 g/day of IPE (2 g twice daily with meals). Subjects were followed for an average of 4.9 years. Then, in the post hoc analysis, the effects of IPE use in patients with recent (<12 months) ACS (*n* = 840) were evaluated.	IPE reduced the incidence of the first primary composite outcome by 37% (*p* = 0.002) and of the first key secondary composite outcome by 36% (*p* = 0.01). Also, IPE lowered CV death and non-fatal MI by 36% (*p* = 0.03) and lowered urgent or emergent revascularization in 44% (*p* = 0.009).	Diarrhea, constipation, dysphagia, belching, AF, and bleeding are treatment-emergent adverse events of any type.
[63]	Phase three B, international, multi-center, prospective, randomized, double-blinded, placebo-controlled, parallel-group trial/United States of America (leading country).	8179 subjects, 59–71 y statin-treated patients with TG levels between ≥135 and <499 mg/dL, LDL-C > 41 and ≤100 mg/dL, and a history of atherosclerosis or DM with additional CV risk factors.	Patients were randomized into the placebo group (*n* = 4000) and IPE group (*n* = 4000), which received 4 g/day of IPE (2 g 2×/day with meals). Subjects were followed for an average of 4.9 years. Then, the post hoc analysis evaluated the CV benefit of IPE associated with different Lp(a) levels.	Lp(a) was significantly related to the first and total major adverse CV events (*p* < 0.0001). IPE significantly reduced the first major adverse CV events in subgroups with ≥50 or <50 mg/dL concentrations.	Diarrhea, constipation, dysphagia, belching, AF, and bleeding are treatment-emergent adverse events of any type.
[64]	Single-center, triple-arm, randomized, controlled, 12-month, open-label trial/Japan.	84 subjects, 13♀, 71♂, medium 68.2 y, and with established coronary artery disease, on statin therapy and LDL-C levels < 100 mg/dL.	Subjects were randomly allocated to one of three groups: the 2 g/day EPA/DHA group, the 4 g/day EPA/DHA group, or the no-treatment group. After 12 months of treatment, atherosclerotic plaque was assessed by CMR.	There was a reduction in the PMR of −0.15 in the 2 g/day and 4 g/day EPA/DHA groups compared to the baseline; in the untreated group, no changes were observed.	Not reported.
[27]	Prospective, 3-month, open-label, randomized study/Canada.	70 subjects, 56♂, 14♀, 56–77 y in statin treatment, with TG ≥ 1.50 and <5.6 mmol/L and either ASCVD or DM2 with additional CV risk factors.	Subjects were randomized to the IPE (4 g/day) group or the usual care group. Then, vascular regenerative cells with ALDHhi were isolated from blood samples collected at baseline and 3-month follow-up visits and characterized using lineage-specific cell surface markers.	IPE increased the mean frequency of ALDHhi side scatter low CD133+ cells (*p* = 0.02), decreased overall ALDHhi side scatter low cell frequency, reduced ROS, and increased ALDHhi side scatter granulocyte precursor cell content.	Gastrointestinal events, *n* = 2, underwent PCI; one was hospitalized for acute kidney injury.

ω-3: omega 3; ACS: acute coronary syndrome; AF: atrial fibrillation/atrial flutter; ALDHhi: high aldehyde dehydrogenase activity; AMR101: Eicosapentaenoic Acid Ethyl Ester; ASCVD: atherosclerotic cardiovascular disease; CABG: coronary artery bypass graft surgery; CCTA: coronary computed tomographic angiography; CMR: cardiovascular magnetic resonance; CV: cardiovascular; DHA: docosahexaenoic acid; DM: diabetes mellitus; DPA: docosapentaenoic acid; EPA: eicosapentaenoic acid; EE: ethyl ester; FFA: free fatty acid; HDL-C: high-density lipoprotein cholesterol; HF: heart failure; HR: hazard ratio/risk ratio; hsCRP: high-sensitivity C-reactive protein; IL: interleukin; IPE: Icosapent Ethyl; LAP: low-attenuation plaque; LDL-C: low-density lipoprotein cholesterol; Lp(a): lipoprotein(a); Lp-PLA_2:_ lipoprotein-associated phospholipase A_2;_ MDCT: multidetector computed tomography; MI: myocardial infarction; *n*-3 PUFA: *n*-3 polyunsaturated fatty acid; ox-LDL-C: oxidized low-density lipoprotein cholesterol; PCI: percutaneous coronary intervention; PMR: plaque-to-myocardium signal intensity ratio; ROS: reactive oxygen species; TG: triglyceride; VLDL: very low-density lipoprotein; WC: waist circumference.

## Data Availability

Not applicable.

## Supplementary Materials

The following supporting information can be downloaded at: https://www.mdpi.com/article/10.3390/ph18040601/s1, Table S1: Descriptive table of the biases of the included randomized clinical trials; Table S2: GRADE analysis.
