# Supplementary material for: Assessing Omega-3 Therapy and Its Cardiovascular Benefits: What About Icosapent Ethyl? A Systematic Review and Meta-Analysis
Source: Pharmaceuticals (Basel). 2025 Apr 20;18(4):601. doi: 10.3390/ph18040601 (PMC12030327; doi:10.3390/ph18040601)
Supplement: Supplementary file 1 [file pharmaceuticals-18-00601-s001.zip › pharmaceuticals-3573902-supplementary.pdf]

Table S1. Descriptive table of the biases of the included randomized clinical trials.

Study	Question focus	Appropriate randomization	Allocation blinding	Double-blind	Losses (>20%)	Prognostics or demographic Characteristics	Outcomes	Intention to treat analysis	Sample calculation	Adequate follow-up
(Ando et al., 1999)	**Yes**	**Yes**	**Yes**	**Yes**	**No**	**Yes**	**Yes**	**No**	**No**	**No**
(Cawood et al., 2010)	**Yes**	**Yes**	**Yes**	**Yes**	**No**	**Yes**	**Yes**	**No**	**Yes**	**No**
(Bays et al., 2012)	**Yes**	**Yes**	**Yes**	**Yes**	**Yes**	**Yes**	**Yes**	**Yes**	**No**	**No**
(Ballantyne et al., 2012)	**Yes**	**Yes**	**Yes**	**Yes**	**No**	**Yes**	**Yes**	**Yes**	**Yes**	**No**
(Alfaddagh et al., 2017)	**Yes**	**Yes**	**No**	**No**	**No**	**Yes**	**Yes**	**Yes**	**Yes**	**Yes**
(Sezai et al., 2019)	**Yes**	**Yes**	**Yes**	**No**	**No**	**Yes**	**Yes**	**No**	**No**	**Yes**
(Miller et al., 2019)	**Yes**	**Yes**	**Yes**	**Yes**	**No**	**Yes**	**Yes**	**Yes**	**Yes**	**No**
(Allaire et al., 2019)	**Yes**	**Yes**	**Yes**	**Yes**	**Yes**	**Yes**	**Yes**	**Yes**	**Yes**	**No**
(Bhatt et al., 2020)	**Yes**	**Yes**	**Yes**	**Yes**	**No**	**Yes**	**Yes**	**Yes**	**Yes**	**Yes**
(Verma et al., 2021)	**Yes**	**Yes**	**Yes**	**Yes**	**No**	**No**	**Yes**	**Yes**	**Yes**	**Yes**
(Budoff et al., 2020)	**Yes**	**Yes**	**Yes**	**Yes**	**No**	**Yes**	**Yes**	**Yes**	**Yes**	**No**
(Budoff et al., 2021)	**Yes**	**Yes**	**Yes**	**Yes**	**No**	**Yes**	**Yes**	**Yes**	**Yes**	**No**
(Peterson et al., 2021)	**Yes**	**Yes**	**Yes**	**Yes**	**No**	**No**	**Yes**	**Yes**	**Yes**	**Yes**

(Peterson et al., 2022)	**Yes**	**Yes**	**Yes**	**Yes**	**No**	**No**	**Yes**	**Yes**	**Yes**	**Yes**
(Gaba et al., 2022)	**Yes**	**Yes**	**Yes**	**Yes**	**No**	**No**	**Yes**	**Yes**	**Yes**	**Yes**
(Maki et al., 2022)	**Yes**	**Yes**	**No**	**No**	**No**	**Yes**	**Yes**	**Yes**	**Yes**	**No**
(Selvaraj et al., 2022)	**Yes**	**Yes**	**Yes**	**Yes**	**No**	**No**	**Yes**	**Yes**	**Yes**	**No**
(Olshansky et al., 2023)	**Yes**	**Yes**	**Yes**	**Yes**	**No**	**No**	**Yes**	**Yes**	**Yes**	**Yes**
(Miller et al., 2023)	**Yes**	**Yes**	**Yes**	**Yes**	**No**	**No**	**Yes**	**Yes**	**Yes**	**Yes**
(Wang et al., 2023)	**Yes**	**Yes**	**Yes**	**Yes**	**No**	**Yes**	**Yes**	**Yes**	**Yes**	**No**
(Sayah et al., 2024)	**Yes**	**Yes**	**Yes**	**Yes**	**No**	**No**	**Yes**	**Yes**	**Yes**	**Yes**
(Szarek et al., 2024)	**Yes**	**Yes**	**Yes**	**Yes**	**No**	**No**	**Yes**	**Yes**	**Yes**	**Yes**
(Nakao et al., 2024)	**Yes**	**Yes**	**No**	**No**	**No**	**Yes**	**Yes**	**Yes**	**Yes**	**No**
(Bakbak et al., 2024)	**Yes**	**Yes**	**No**	**No**	**No**	**Yes**	**Yes**	**Yes**	**Yes**	**No**

Table S2. GRADE analysis.

Author(s):
Question: Icopesant compared to controle for Perfil lipídico
Setting:
Bibliography:

Certainty assessment							№ of patients		Effect		Certainty	Importance
№ of studies	Study design	Risk of bias	Inconsistency	Indirectness	Imprecision	Other considerations	Icopesant	controle	Relative (95% CI)	Absolute (95% CI)		
**Total cholesterol (follow-up: mean 12 months; assessed with: mg/dL; Scale from: 0 to 150)**												
2	randomised trials	serious[a,b]	not serious	not serious	serious[c]	none	56	64	-	mean **1.91 mg/dL lower** (12.09 lower to 8.27 higher)	⨁⨁◯◯ Low[a,b,c]	CRÍTICO
**HDL (follow-up: mean 12 months; assessed with: mg/dL; Scale from: 0 to 50)**												
2	randomised trials	serious[a,b]	not serious	not serious	serious[c]	none	56	64	-	mean **0.56 mg/dL lower** (4.03 lower to 2.9 higher)	⨁⨁◯◯ Low[a,b,c]	CRÍTICO
**LDL (follow-up: mean 12 months; assessed with: mg/dL; Scale from: 0 to 90)**												
2	randomised trials	serious[a,b]	not serious	not serious	serious[c]	none	56	64	-	mean **3.77 mg/dL lower** (11.81 lower to 4.27 higher)	⨁⨁◯◯ Low[a,b,c]	CRÍTICO
**Triglycerides (follow-up: mean 12 months; assessed with: mg/dL; Scale from: 0 to 90)**												
2	randomised trials	serious[a,b]	not serious	not serious	serious[c]	none	56	64	-	mean **3.77 mg/dL lower** (11.81 lower to 4.27 higher)	⨁⨁◯◯ Low[a,b,c]	CRÍTICO

CI: confidence interval

Explanations

a. Inadequate follow up
b. Losses (>20%)
c. High CI95%
